# Noble gas neuroprotection: xenon and argon protect against hypoxic–ischaemic injury in rat hippocampus *in vitro* via distinct mechanisms

**DOI:** 10.1016/j.bja.2019.07.010

**Published:** 2019-08-27

**Authors:** Mariia Koziakova, Katie Harris, Christopher J. Edge, Nicholas P. Franks, Ian L. White, Robert Dickinson

**Affiliations:** 1 Anaesthetics, Pain Medicine and Intensive Care Section, Department of Surgery and Cancer, Imperial College London, London, UK; 2 Department of Life Sciences, Imperial College London, London, UK; 3 Department of Anaesthetics, Royal Berkshire Hospital NHS Foundation Trust, London Road, Reading, UK; 4 Department of Anaesthetics, St Peter's Hospital, Chertsey, UK; 5 Royal British Legion Centre for Blast Injury Studies, Department of Bioengineering, Imperial College London, London, UK

**Keywords:** acute brain injury, carbon monoxide poisoning, hypoxic–ischaemic encephalopathy, out-of-hospital cardiac arrest, neuroprotection, noble gases, stroke

## Abstract

**Background:**

Noble gases may provide novel treatments for neurological injuries such as ischaemic and traumatic brain injury. Few studies have evaluated the complete series of noble gases under identical conditions in the same model.

**Methods:**

We used an *in vitro* model of hypoxia–ischaemia to evaluate the neuroprotective properties of the series of noble gases, helium, neon, argon, krypton, and xenon. Organotypic hippocampal brain slices from mice were subjected to oxygen-glucose deprivation, and injury was quantified using propidium iodide fluorescence.

**Results:**

Both xenon and argon were equally effective neuroprotectants, with 0.5 atm of xenon or argon reducing injury by 96% (*P*<0.0001), whereas helium, neon, and krypton were devoid of any protective effect. Neuroprotection by xenon, but not argon, was reversed by elevated glycine.

**Conclusions:**

Xenon and argon are equally effective as neuroprotectants against hypoxia–ischaemia in vitro, with both gases preventing injury development. Although xenon's neuroprotective effect may be mediated by inhibition of the *N*-methyl-d-aspartate receptor at the glycine site, argon acts *via* a different mechanism. These findings may have important implications for their clinical use as neuroprotectants.

**Editor's key points**

• Noble gases have shown neuroprotective effects in experimental models of cerebral ischaemia.
• An *in vitro* model of cerebral ischaemia was used to compare the neuroprotective efficacy of the full series of noble gases.
• Whereas xenon and argon were similarly neuroprotective, helium, neon, and krypton were without a protective effect.
• Reversal of neuroprotection by xenon, but not by argon, by elevated glycine suggests distinct protective mechanisms.
• Further translational studies to evaluate these two noble gases as neuroprotectants are warranted by these findings.

Neurological injuries resulting from hypoxia–ischaemia are leading causes of morbidity and mortality worldwide.^1^, ^2^, ^3^ Hypoxic–ischaemic brain injury has a variety of aetiologies including stroke, cardiac arrest, neonatal hypoxic–ischaemic encephalopathy (HIE), drowning and exposure to asphyxiant gases and carbon monoxide. Many who survive a hypoxic–ischaemic brain injury have persisting disability, with long-term care and rehabilitation costs.^4^ Treatment options are limited to thrombolytic drugs and clot removal for ischaemic stroke, and therapeutic cooling (or hypothermia) for cardiac arrest and neonatal HIE. Currently, there are no clinically proven treatments specifically targeted at preventing or limiting neuronal death resulting from ischaemia.

There is a need to develop neuroprotective treatments for hypoxic–ischaemic brain injury. Currently there is interest in the noble gases as novel treatments for ischaemic and traumatic brain injury.^5^, ^6^, ^7^, ^8^, ^9^ Attention has focused on xenon, which has already undergone clinical trials for HIE^10^, ^11^, ^12^ and out-of-hospital cardiac arrest,^13^, ^14^ but there is also interest in the use of argon and helium, which have been evaluated in *in vitro* and *in vivo* models.^15^, ^16^, ^17^, ^18^, ^19^, ^20^ Neuroprotection by particular noble gases has been reported under different conditions.^7^, ^21^, ^22^, ^23^, ^24^, ^25^ Few studies, however, have evaluated neuroprotection by krypton or neon,^26^, ^27^ and investigation of the whole series of noble gases in hypoxic–ischaemic brain injury under the same conditions has been limited to dissociated cell cultures.^27^ We report the neuroprotective efficacy of helium, neon, argon, krypton, and xenon under identical conditions using organotypic hippocampal brain-slice cultures subjected to oxygen-glucose deprivation (OGD), an experimental model of cerebral ischaemia. We tested the hypothesis that the *N*-methyl-d-aspartate (NMDA) receptor glycine site is involved in noble gas neuroprotection against hypoxic–ischaemic brain injury *in vitro*.

## Methods

Unless otherwise stated, chemicals were obtained from Sigma-Aldrich Ltd (Gillingham, Dorset, UK). All gases were obtained from BOC Ltd (Guildford, Surrey, UK); pure noble gases were N5.0 grade (99.999%).

### Hippocampal organotypic slices

Experiments were performed in compliance with the Animal Welfare and Ethical Review Body of Imperial College London and the Animals (Scientific Procedures) Act of 1986. Animals (pups and their dams) were housed in individually ventilated cages in a pathogen-free facility in a 12:12 h light–dark cycle (7:00 am–7:00 pm light) at 22°C with *ad libitum* access to food and water. Animals were checked at least once daily. Organotypic hippocampal slice cultures were prepared as described^24^, ^26^, ^28^, ^29^ from male and female 7-day-old C57BL/6 mouse pups (Harlan Ltd, Bicester, Oxfordshire, UK). Briefly, after euthanasia, brains were removed and placed in ice-cold ‘preparation’ medium that contained Gey's balanced salt solution, 33 mM d-glucose (Fisher Scientific, Loughborough, Leicestershire, UK) and 1% antibiotic–antimycotic suspension. The hippocampi were removed, and 400 μm thick transverse slices were prepared using a McIllwain tissue chopper. Slices were transferred into ice-cold preparation medium, gently separated and then placed on tissue culture inserts (Millicell-CM; Millipore Corporation, Carrigtwohill, Co. Cork, Ireland) that were inserted into a six-well tissue culture plate. The wells contained ‘growth’ medium consisting of 50% (v:v) Minimal Essential Medium Eagle, 25% Hank's balanced salt solution, 25% inactivated horse serum, 2 mM l-glutamine, 32 mM d-glucose, and 1% antibiotic–antimycotic suspension. Slices were incubated at 37°C in a 95% air:5% CO_2_ humidified atmosphere. The growth medium was changed every 3 days. Experiments were carried out after 14 days in culture. Cell culture inserts containing four to seven slices were randomly assigned to sham, OGD control, or OGD noble gas treatment groups.

### Oxygen-glucose deprivation and hyperbaric gas chamber

**Fig 1:**
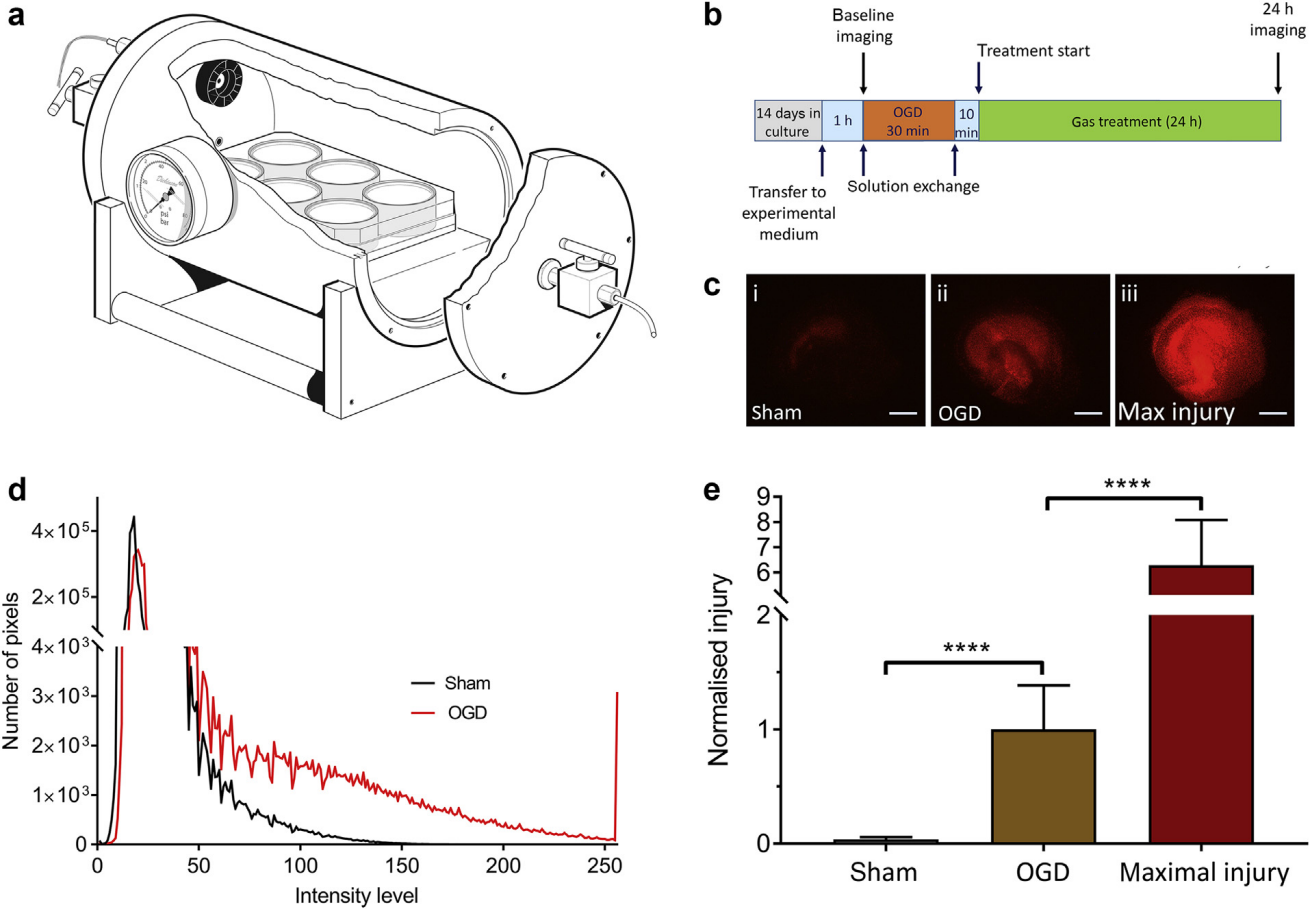
(a) Diagram of the chamber used for oxygen-glucose deprivation (OGD) and gas treatment. Organotypic hippocampal slice cultures in six-well cell-culture dishes were placed in the chamber. A small fan (shown in black) ensured mixing of the gases. (b) Schematic showing the experimental timeline. (c) Typical propidium iodide fluorescence images at of slices (i) sham, (ii) OGD, and (iii) maximal injury. Scale bars=500 μm. (d) Intensity histogram of slices from sham (black) and OGD (red) groups. (e) Quantification of injury in sham (white bar), OGD (brown bar), and maximal injury (dark red bar) slices at 24 h after injury or sham procedure. Slices were exposed to control gas (75% N_2_:20% O_2_:5% CO_2_) with 0.5 atm helium for 24 h after OGD or sham procedure. The intensity histograms are the mean of 10 (sham) and 25 (OGD) slices. Pixel numbers have been normalised to the median of the OGD slices. Bars are median values, error bars are the 95% confidence interval. *n*=191, sham; *n*=326, OGD, *n*=125 max injury. ****P<0.0001, Kruskal–Wallis test with Dunn's correction for multiple comparisons.

The growth medium was changed to serum-free ‘experimental’ medium consisting of 75% Minimal Essential Medium Eagle, 25% Hank's balanced salt solution, 2 mM l-glutamine, 33 mM d-glucose, 1% antibiotic–antimycotic suspension, and 4.5 μM propidium iodide (PI). One hour after transfer to experimental media, slices were imaged to assess viability before OGD. Typically, slices exhibited very little PI fluorescence, an indicator of healthy slices. A small number of slices were excluded from further analysis because they failed to meet objective viability criteria at this time point (*t*=0); either there were regions of dense staining, or there were more than 20 pixels at intensity levels above 80, or tissue fragments were visible, indicating compromised viability, presumably as a result of mechanical damage during slice preparation. Immediately after initial imaging, experimental medium was exchanged for ‘OGD medium’, 120 mM NaCl, 5 mM KCl, 1.25 mM NaH_2_PO_4_, 2 mM MgSO_4_, 2 mM CaCl_2_, 25 mM NaHCO_3_, 10 mM sucrose, 20 mM HEPES, pH 7.25 or ‘sham medium,’ which had the same composition, except that sucrose was replaced with 10 mM d-glucose. OGD medium was deoxygenated before use by bubbling for 45 min at 50 ml min^−1^ with 95% N_2_:5% CO_2_, in a Dreschel bottle using a fine-sintered glass bubbler and filter-sterilised using a 0.2 μm filter. Sham medium was treated in the same way except it was bubbled with 20% O_2_:75% N_2_:5% CO_2_. After solution exchange, culture dishes were transferred to a small chamber (Fig. 1a) that contained a high-speed fan for rapid gas mixing. The chamber was housed in an incubator at 37°C. The chamber (gas volume 0.925 L) was flushed with humidified gas (95% N_2_:5% CO_2_ or 20% O_2_:75% N_2_:5% CO_2_) for 5 min at 5 L min^−1^ ensuring better than 99.99% gas replacement. After flushing, the chamber was sealed for a set period of 30 min, constituting the duration of OGD (or sham treatment).

After the period of OGD, slices were removed from the chamber and medium was replaced with experimental medium (in experiments with added glycine [100 μM], this was added for the first time at this stage). Slices were returned to the chamber which was flushed with 20% O_2_:75% N_2_:5% CO_2_ as before and sealed. In the helium experiments at 1.0 atm the chamber was flushed with 20% O_2_:75% He:5% CO_2_. In experiments with xenon, krypton, argon, and neon, after flushing with 20% O_2_:75% N_2_:5% CO_2_ an additional 0.5 atm of noble gas was added after sealing the chamber, with helium used as control for the effects of pressure. Treatment with noble gases was started 10 min after OGD. For all gas mixtures (except during OGD), the partial pressures of oxygen and carbon dioxide were fixed at 0.2 and 0.05 atm, respectively. During OGD, the partial pressures were 0.95 atm nitrogen and 0.05 atm carbon dioxide. The chamber fan was left on for 5 min to achieve mixing of gases. After 24 h in the chamber, slices were imaged using a fluorescent microscope (see section ‘Quantifying cell injury’). The experimental timeline is shown in Fig. 1b.

### Quantifying cell injury

PI only enters cells with compromised cellular membranes and becomes fluorescent after binding to nucleic acids, allowing quantification of cell injury.^30^, ^31^, ^32^ The PI assay does not distinguish between different cell types or grey and white matter, as would be possible with histopathology, but PI has the advantage in that real-time quantification of injury can take place in the same slices at different time points (in this case, the viability assessment at *t*=0 h before injury and at *t*=24 h after OGD or sham procedure). An epifluorescence microscope (Nikon Eclipse 80; Kingston upon Thames, Surrey, UK), with a low-power (2×) objective was used to quantify PI fluorescence. A digital video camera and software (Micropublisher 3.3 RTV camera and QCapture Pro software; Qimaging Inc, Surrey, BC, Canada) were used to capture the images. Image intensity analysis of the red channel was performed using ImageJ software,^33^ with the distribution of intensities plotted as a histogram over 256 intensity levels. Uninjured sham slices under control conditions, incubated in the chamber for 24 h at 37°C with 20% O_2_:75% N_2_:5% CO_2_, showed little PI fluorescence (Fig. 1c[i]) compared with OGD injured slices (Fig. 1c[ii]) that exhibited bright PI fluorescence. In order to determine the relative magnitude of the OGD injury we determined maximal cell death by incubating some slices in 70% ethanol overnight at 4°C (Fig. 1c[iii]). To quantify the injury we integrated the number of pixels above a threshold of 100, which provides a robust quantitative measurement of cell injury (Fig. 1d).^24^ Absolute pixel values were normalised to the median value of the control OGD slices (Fig. 1e).

### Statistical analysis

Data were tested for normality using the Shapiro–Wilk test and found to be non-normal. Results are shown as median values with error bars representing 95% confidence intervals. We assessed significance using the Kruskal–Wallis test with Dunn's correction for multiple comparisons. A *P*-value of <0.05 was taken to indicate a significant difference between groups. Statistical tests were performed using GraphPad Prism v 7.04 (GraphPad Inc., La Jolla, CA, USA).

## Results

### Oxygen-glucose deprivation results in sub-maximal injury

To determine the relative intensity of our OGD injury we compared uninjured sham slices with slices subjected to OGD and slices subjected to maximal injury. Compared with uninjured shams, slices subjected to OGD exhibited a bright PI fluorescence, which was sub-maximal (Fig. 1c and d). Injury in the OGD slices was greater than sham and less than maximal injury (Fig. 1e).

### Helium has no effect on hypoxic–ischaemic injury

**Fig 2:**
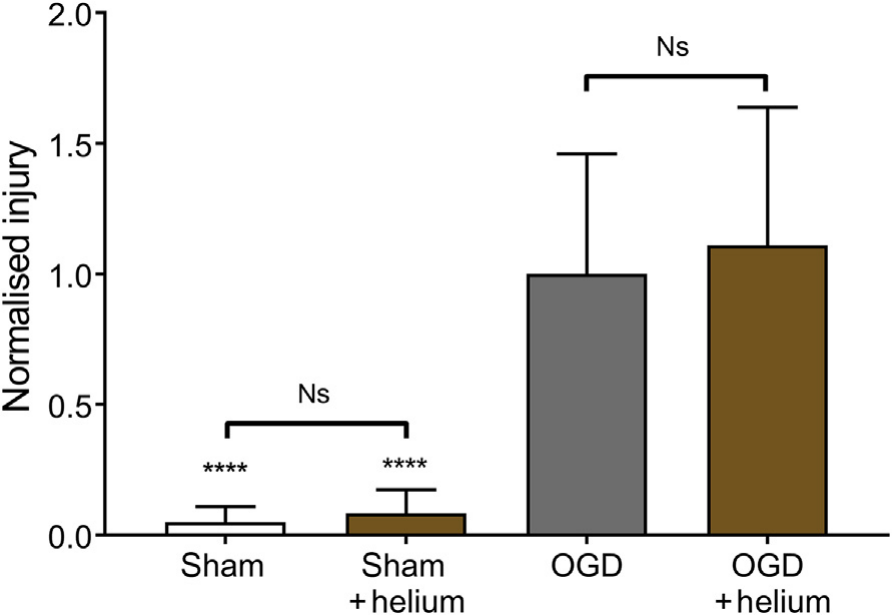
Helium at atmospheric pressure has no effect after injury or sham procedure. Slices were exposed to either control gas (75% N_2_:20% O_2_:5% CO_2_) or helium (75% He:20% O_2_:5% CO_2_) for 24 h after OGD or sham procedure. Pixel numbers have been normalised to the median of the control OGD slices. Bars are median values, error bars are the 95% confidence interval. *n*=31, sham; *n*=35, sham helium; *n*=54, OGD; *n*=46, helium OGD. ****p<0.0001 compared with OGD, Kruskal–Wallis test with Dunn's correction for multiple comparisons. Ns, not significant; OGD, oxygen-glucose deprivation.

We determined the effect of 1.0 atm helium (75% He:20% O_2_:5% CO_2_) on sham and OGD slices (Fig. 2). There was no significant difference between sham slices with or without helium. Injury was low in sham slices, with median values 4.9% and 8.3% of the median value of the control OGD in the absence and presence of helium, respectively. After OGD, injury developed significantly (*p* < 0.0001) at 24 h compared with shams, in both the absence and presence of helium. However, there was no significant difference between OGD slices treated with helium and control OGD slices treated with 75% N_2_:20% O_2_:5% CO_2_.

### Xenon and argon prevent hypoxic–ischaemic injury, whereas krypton and neon have no effect

**Fig 3:**
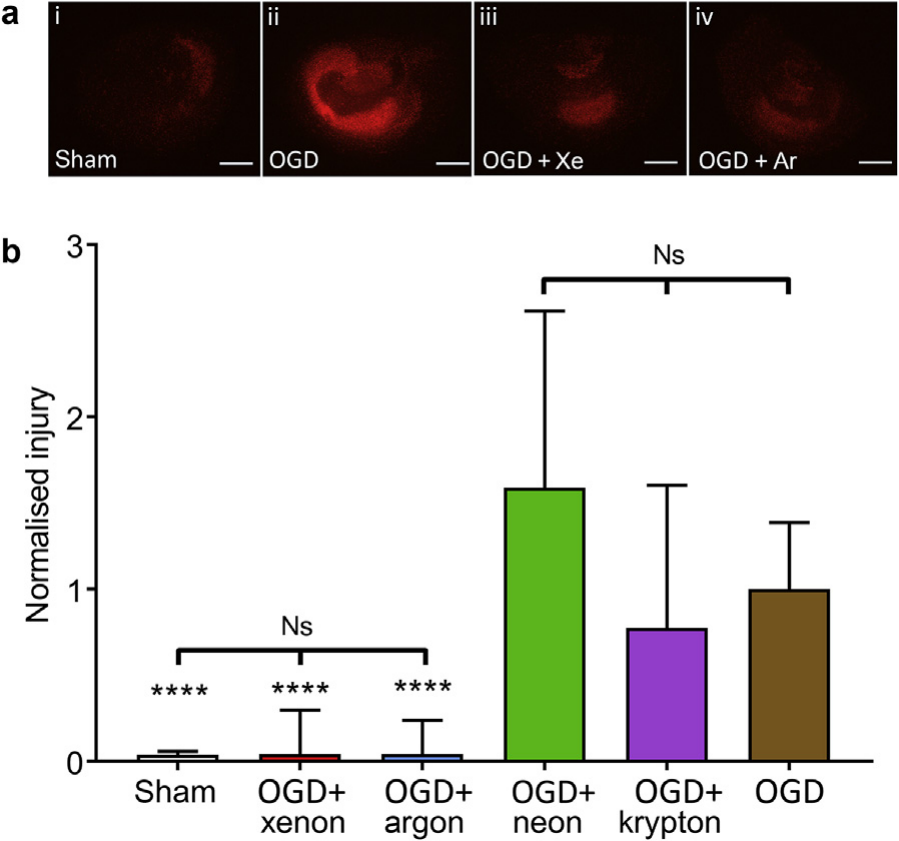
Xenon and argon prevent after OGD injury whereas other noble gases have no protective effect. (a) Typical propidium iodide fluorescence images of slices (i) sham, (ii) OGD + 0.5 atm helium, (iii) OGD + 0.5 atm xenon, (iv) OGD + 0.5 atm argon. Scale bars are 500 μm. (b) Quantification of injury at 24 h, in sham (white bar), OGD + 0.5 atm xenon (red bar), OGD + 0.5 atm argon (blue bar), OGD + 0.5 atm neon (green bar), OGD + 0.5 atm krypton (purple bar), OGD control (brown bar). OGD control and sham slices were exposed to 0.5 atm helium. All slices were also exposed to 1.0 atm control gas (75% N_2_:20% O_2_:5% CO_2_) with total partial pressure 1.5 atm. Pixel numbers have been normalised to the median of the OGD + 0.5 atm helium slices. Bars are median values, error bars are the 95% confidence interval. *n*=191, sham; *n*=95, OGD + xenon; *n*=52, OGD + argon; *n*=89, OGD + neon; *n*=108, OGD + krypton; *n*=326, OGD control. ****P<0.0001 compared with OGD, Kruskal–Wallis test with Dunn's correction for multiple comparisons. Ns, not significant; OGD, oxygen-glucose deprivation.

As helium was without effect, we investigated the effect of 0.5 atm of the noble gases xenon, krypton, argon, and neon on OGD injury (Fig. 3). As these experiments used mild hyperbaric conditions, we used 0.5 atm helium in the control OGD to control for any effects of pressure. Sham slices exhibited very little injury (Fig. 3a[i]) compared with control OGD (Fig. 3a[ii]), whereas treatment with xenon (Fig. 3a[iii]) or argon (Fig. 3a[iv]) after OGD reduced injury. Xenon and argon were equally effective at reducing OGD injury, both reducing injury significantly (*p* < 0.0001) by 96% (Fig. 3b). The OGD slices treated with xenon and argon were not significantly different to each other or to the uninjured sham group (Fig. 3b). Thus both of these noble gases can prevent injury development in this *in vitro* model. We found that krypton and neon were without significant effect on OGD injury (Fig. 3b).

### Glycine reverses the protective effect of xenon, but not of argon, against hypoxic–ischaemic injury

**Fig 4:**
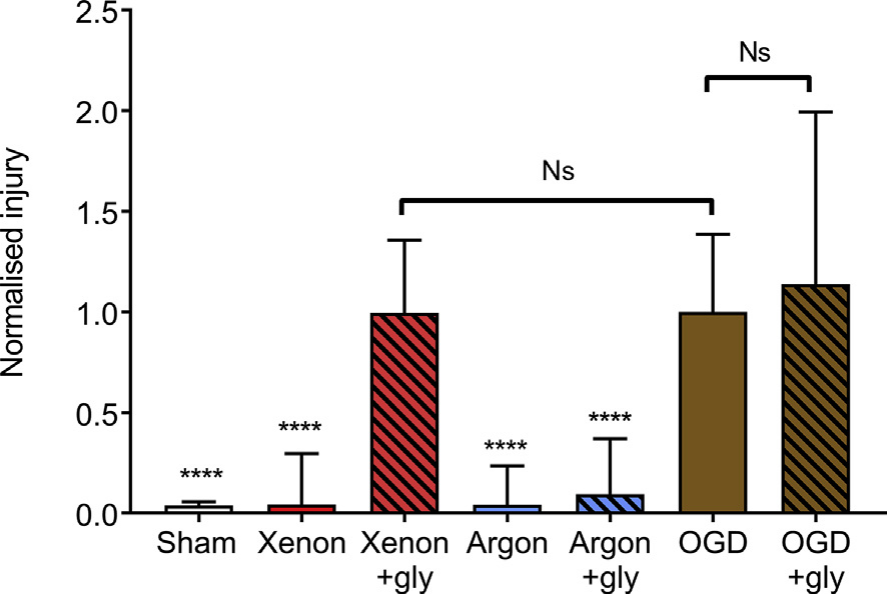
Neuroprotection by xenon but not argon is reversed by elevated glycine. The addition of 100 μM glycine has no significant effect on the control OGD injury, but reverses the protective effect of 0.5 atm xenon. Quantification of injury at 24 h in sham (white bar), OGD + 0.5 atm xenon (red bar), OGD + 0.5 atm xenon + glycine (red hatched bar), OGD + 0.5 atm argon (blue bar), OGD + 0.5 atm argon + glycine (blue hatched bar), OGD control (brown bar), OGD control + glycine (brown hatched bar). OGD control and sham slices were exposed to 0.5 atm helium. All slices were also exposed to 1.0 atm control gas (75% N_2_:20% O_2_:5% CO_2_) with total partial pressure 1.5 atm. Pixel numbers have been normalised to the median of the OGD control slices. Bars are median values, error bars are the 95% confidence interval. *n*=191, sham; *n*=95, OGD + xenon; *n*=91, OGD + xenon + glycine; *n*=52, OGD + argon; *n*=48, OGD + argon + glycine; *n*=326, OGD control; *n*=69, OGD control + glycine. ****P<0.0001 compared with OGD, Kruskal–Wallis test with Dunn's correction for multiple comparisons. Ns, not significant; OGD, oxygen-glucose deprivation; gly, glycine.

In order to determine the role of the NMDA receptor in the protective effect of xenon and argon, we investigated the effect of glycine on neuroprotection by these noble gases. The addition of 100 μM glycine had no significant effect on control OGD injury with helium (Fig. 4). The protective effect of argon was unaffected by addition of glycine, with a 91% reduction with glycine compared with 96% reduction without glycine. In contrast, addition of glycine completely reversed the protective effect of xenon, with a 96% reduction in injury without and a 0.4% reduction in injury with added glycine. This is consistent with xenon neuroprotection being mediated by the NMDA receptor glycine binding site. These findings indicate that xenon and argon provide neuroprotection against hypoxic–ischaemic injury by different mechanisms.

## Discussion

### Oxygen-glucose deprivation model

Organotypic hippocampal slice cultures (OHSCs) were subjected to OGD, with injury quantified by PI fluorescence. This preparation retains a variety of cell types (e.g. different types of neurones and glia) with cellular organisation and synaptic connectivity similar to *in vivo*,^34^, ^35^ and is widely used as an intermediate between dissociated cell cultures and whole-animal models.^20^, ^24^, ^26^, ^28^, ^36^, ^37^, ^38^, ^39^, ^40^ We chose 30 min as the duration of OGD because we previously showed that this produced a reliable and robust injury.^24^ OGD results in a diffuse global injury, and the injury produced by 30 min OGD was sub-maximal. The OHSC model we used has advantages and limitations. An *in vitro* model allows us to control the slice environment. Organotypic brain slice cultures exposed to OGD are a widely used model of cerebral hypoxia–ischaemia,^41^, ^42^, ^43^ and *in vitro* OGD causes disruption of cellular function that is similar to hypoxia–ischaemia *in vivo*.^44^, ^45^, ^46^ We measured cell death and neuroprotection in the hippocampal slice as a whole in order to avoid subjectivity associated with precisely defining the boundaries of CA1, CA3, and dentate gyrus. In humans with ischaemic brain injury, hippocampal sub-regions exhibit differential sensitivity, with CA1 being particularly vulnerable.^47^ In our study, we observed qualitatively that the CA1 region appeared more sensitive to ischaemic injury, in agreement with clinical data and previous *in vitro* studies,^24^, ^48^ and this likely reflects the density of NMDA receptors. Nevertheless, xenon and argon appeared to reduce injury to a similar degree in different hippocampal areas as observed for other neuroprotective drugs.^24^, ^26^, ^42^, ^43^, ^49^

### Lack of effect of helium

There are few studies of helium as a neuroprotectant, and these have produced contradictory results. Helium was found to be neuroprotective in a rat model of ischaemic brain injury; however, this was shown not to be a pharmacological effect but because of hypothermia resulting from breathing helium at room temperature (because of the high thermal conductivity of helium).^5^, ^21^, ^50^ In an *in vitro* OGD model in isolated cell cultures, a detrimental effect of helium was reported.^27^ We previously showed that mild hyperbaric helium (0.5 atm partial pressure at 1.5 atm) had no effect on OGD injury in hippocampal slices.^24^ In the current study, we investigated the effects of 0.75 atm helium under normobaric conditions (1 atm) and found that normobaric helium had no effect on uninjured sham slices or on OGD slices after 24 h of treatment. Our treatment is given inside a temperature-controlled incubator at 37°C; hence, we can exclude the effect of hypothermia as found in the *in vivo* studies. In our OHSC model at 37°C, helium is devoid of any observable effect against OGD.

### Xenon and argon are equally effective as neuroprotectants, whereas other noble gases are without effect

In contrast to helium, both xenon and argon resulted in significant neuroprotection. Both gases prevented injury development; OGD slices treated with 0.5 atm xenon or 0.5 atm argon were not significantly different to uninjured sham slices. Interestingly, we found that xenon and argon were equally effective in the OGD model, in contrast to an *in vitro* model of traumatic brain injury where argon was less effective than xenon.^26^ The reasons why argon appears to be as effective as xenon in this ischaemic injury model are not clear, but this suggests that different secondary injury mechanisms may be involved in ischaemic and traumatic brain injury. The comparable efficacy of xenon and argon we observe *in vitro* does not necessarily mean that similar long-term functional improvements will be observed for these gases *in vivo*. The relative efficacy of xenon and argon on clinically relevant long-term functional outcomes after hypoxia–ischaemia *in vivo* remains to be determined. Krypton and neon were without any protective effect, consistent with findings on isolated cell cultures subjected to OGD and OHSCs that had experienced a traumatic insult.^26^, ^27^

### Neuroprotection by xenon, but not argon, involves NMDA receptor inhibition at the glycine site

Xenon inhibits the NMDA receptor by competing for the binding of the co-agonist glycine, and xenon inhibition can be prevented by elevating glycine concentrations.^51^, ^52^ In the current study we found that addition of glycine had no effect on the control OGD injury. The simplest explanation for this observation is that the concentration of endogenous glycine is just below saturating on the concentration–effect curve. However, the neuroprotective effect of xenon was completely reversed by the addition of glycine, consistent with inhibition of the NMDA receptor glycine site mediating xenon's protective effect. In contrast, addition of glycine had no effect on neuroprotection by argon, indicating that argon acts *via* a different mechanism. The reversal of neuroprotection by xenon but not argon with added glycine is consistent with what we observed in an *in vitro* model of traumatic brain injury.^26^ Xenon acts at other targets, such as the two pore-domain potassium channel TREK-1 and the ATP-sensitive potassium (K-ATP) channel, but has no effect on N-type calcium channels^53^, ^54^, ^55^; however, our findings indicate that inhibition of the NMDA receptor glycine site is likely to play a major role in the neuroprotective effect of xenon.^24^, ^26^, ^51^, ^52^, ^56^, ^57^ The targets mediating the neuroprotective effect of argon are less clear; we have shown that argon does not inhibit NMDA receptors or activate TREK-1 channels.^26^ Nevertheless, other *in vitro* and *in vivo* studies with argon have identified activation of signalling pathways involving MEK-ERK 1/2 and PI3K/AKT, with up-regulation of heme-oxygenase-1.^17^, ^58^, ^59^ A recent study has also identified Nrf2 and the mammalian target of rapamycin (mTOR) signalling pathway as targets for argon.^60^ Although these studies clearly identify changes in these signalling pathways after argon treatment, it is not clear whether argon is acting on the upstream targets of these pathways.

### Clinical relevance of our findings

There is currently much interest in the clinical use of noble gases as neuroprotectants in ischaemic brain injuries. Xenon is licenced for use as a general anaesthetic and has already undergone clinical trials in neonatal HIE, ischaemic brain damage after cardiac arrest, coronary artery bypass graft surgery, and orthopaedic surgery in the elderly.^10^, ^11^, ^13^, ^14^, ^61^, ^62^, ^63^ The most recent study in ischaemic brain injury in cardiac arrest patients found reduced early white-matter damage in the xenon-treated group,^14^ and a larger multi-centre study is underway. Relative to the other inert gases, xenon is more expensive and is used in closed or semi-closed circuits to conserve gas. There has been interest in evaluating other less expensive noble gases as neuroprotectants that could be given in open circuits. Helium and oxygen mixtures have been used medically for respiratory conditions such as asthma and chronic obstructive pulmonary disease (COPD) in adults and bronchiolitis and croup in children, but systematic reviews conclude that the currently available evidence does not support its use in these conditions.^64^, ^65^, ^66^, ^67^ Our finding that helium has no effect on hypoxic–ischaemic injury *in vitro* is consistent with a lack of pharmacological effect of helium at normobaric pressures. The finding that helium has neuroprotective properties in animal models of hypoxia–ischaemia *via* the *physical* mechanism of inducing cooling^5^, ^21^ is an interesting observation, but more straightforward and controllable techniques for therapeutic cooling are available. Our finding that argon is neuroprotective agrees with a large body of *in vitro* and *in vivo* evidence.^7^, ^18^, ^68^ Interestingly, in this OGD model we found 0.5 atm argon to be as effective as 0.5 atm xenon, in contrast to our earlier work with an *in vitro* model of traumatic brain injury where argon was less effective than xenon. Argon has been shown not to affect cerebral circulation in humans,^69^ and there are proposals to evaluate argon as a neuroprotectant in patients.^6^ One obstacle, perhaps, to its clinical use is an unambiguous identification of its mode of action but, on the other hand, it is the most abundant of the noble gases and the cheapest to produce. On a positive note, the fact that argon and xenon do not act by the same mechanism means that combinations of these gases may have a synergistic effect, or that argon and xenon are effective treatments for different forms of ischaemic neurological injury. The observation that in a mouse model of traumatic brain injury, xenon is able to prevent development of very late-onset traumatic brain injury-related memory deficits with reduced white matter loss in the corpus callosum is of great interest,^9^ but it remains to be seen if this will translate into similar findings in humans.

In conclusion our findings that argon and xenon are equally effective neuroprotective in hypoxic–ischaemic injury but act *via* different mechanisms will prompt further translational studies to evaluate these two noble gases as neuroprotectants, either singly or in combinations.

## Authors' contributions

Study design/planning: RD, KH, ILW.

Study conduct: MK, KH.

Data analysis: RD, CJE, MK, KH.

Drafting of paper: RD, CJE.

Revision of and responsibility for paper contents: all authors.
